# Supplementary material for: Factor productivity impacts of climate change and estimating the technical efficiency of cereal crop yields: Evidence from sub-Saharan African countries
Source: PLoS One. 2024 Nov 21;19(11):e0310989. doi: 10.1371/journal.pone.0310989 (PMC11581406; doi:10.1371/journal.pone.0310989)
Supplement: S1 Appendix — (DOCX) [file pone.0310989.s001.docx]

**S1: Appendix**

Table 1: The panel unit root test using the fisher type stationary test

| **Variables** |  | statistics | p-value |
| --- | --- | --- | --- |
| CO2 emissions | Inverse chi-squared(34) p* | 462.8604 | (0.0000) *** |
|  | Inverse normal (z) | -19.4282 | (0.0000)*** |
|  | Inverse logit t(89) (L) | -31.1235 | (0.0000) *** |
|  | Modified inv. chi-squared (Pm) | 52.0070 | (0.0000) *** |
| Cereal yield k.g/ hectare | Inverse chi-squared(34) p* | 687.3200 | (0.0000)*** |
|  | Inverse normal (z) | -24.2702 | (0.0000) *** |
|  | Inverse logit t(89) (L) | -46.2165 | (0.0000) *** |
|  | Modified inv. chi-squared (Pm) | 79.2267 | (0.0000) *** |
| Land Productivity | Inverse chi-squared(34) p* | 558.0353 | (0.0000)*** |
|  | Inverse normal (z) | -21.4623 | (0.0000)*** |
|  | Inverse logit t(89) (L) | -37.5232 | (0.0000)*** |
|  | Modified inv. chi-squared (Pm) | -37.5232 | (0.0000)*** |
| Labor Productivity | Inverse chi-squared(34) p* | 588.3153 | (0.0000)*** |
|  | Inverse normal (z) | -22.1465 | (0.0000)*** |
|  | Inverse logit t(89) (L) | -39.5593 | (0.0000)*** |
|  | Modified inv. chi-squared (Pm) | 67.2206 | (0.0000)*** |
| Fertilizer productivity | Inverse chi-squared(34) p* | 569.2010 | (0.0000)*** |
|  | Inverse normal (z) | -21.3767 | (0.0000)*** |
|  | Inverse logit t(89) (L) | -38.2738 | (0.0000)*** |
|  | Modified inv. chi-squared (Pm) | 64.9027 | (0.0000)*** |
| Agricultural methane emissions | Inverse chi-squared(34) p* | 480.7737 | (0.0000)*** |
|  | Inverse normal (z) | -19.8378 | (0.0000)*** |
|  | Inverse logit t(89) (L) | -32.3280 | (0.0000)*** |
|  | Modified inv. chi-squared (Pm) | 54.1793 | (0.0000)*** |
|  | Inverse chi-squared(34) p* | 713.5817 | (0.0000)*** |
|  | Inverse normal (z) | -24.8907 | (0.0000)*** |
|  | Inverse logit t(89) (L) | -47.9824 | (0.0000)*** |
|  | Modified inv. chi-squared (Pm) | 82.4114 | (0.0000)*** |

**Source: own computation STATA** 17
