# Supplementary material for: Factor productivity impacts of climate change and estimating the technical efficiency of cereal crop yields: Evidence from sub-Saharan African countries
Source: PLoS One. 2024 Nov 21;19(11):e0310989. doi: 10.1371/journal.pone.0310989 (PMC11581406; doi:10.1371/journal.pone.0310989)
Supplement: Attachment — Submitted filename: Response to reviewer.docx [file pone.0310989.s003.docx]

**Author response letter**

PONE-D-24-08690

Factor productivity impacts of climate change and estimating the technical efficiency of cereal crop yields: Evidence from sub-Saharan African countries

 We thank the editors and reviewers for your efforts and time spent providing constructive comment. We believe the criticisms are valid and will help us to improve the originality of the study. The following are the line to line responses to editors and reviewers for comments requested to be modifications.

**Response to Editor:**

1. The work is amended based on the guidelines and the PLOS ONE journal style template, which are available at

<https://journals.plos.org/plosone/s/file?id=wjVg/PLOSOne_formatting_sample_main_body.pdf> and  [https://journals.plos.org/plosone/s/file?id=ba62/PLOSOne_formatting_sample_title_authors_affiliations.pdf](https://journals.plos.org/plosone/s/file?id=ba62/PLOSOne_formatting_sample_title_authors_affiliations.pdf)

2. We rewrite and read to correct errors in language, spelling, and grammar. It was also corrected and grammatical verified by our friend Mr. Yibeltal Walle, a language instructor and researcher at Debre Tabor University. However, we cannot access the service of the American Journal Experts (AJE).
3. The authors will provide the data when requested by the journal. The data deposited from (<https://databank.worldbank.org/source/world-development-indicators>).
4. We tried to construct the manuscript based on the template, thus we included and cited all figures and tables. Table 5 is referenced in the text. The comments, all of which were good, have enriched and changed the work significantly.

**Response to Reviewer #1**

**Dear Reviewer One.** We thank you for sharing your knowledge and skills, and your suggestions have greatly aided us in rewriting and enriching our work.

1. The abstract has been rewritten by considering the introduction, methodology, objectives, findings, conclusion, and policy implications.
2. Additionally, it was suggested that the study's paper be rewritten and expanded to include unique content. We are dedicated to answering your question, and the gap is rewritten as follows: Studies like [5], [24], [27]–[29] focused on the impact of climate change on crop yields whereas [23], [36], [39], [40] investigated on climate change impact on technical efficiency. However, they failed to investigate the impact of climate change on agricultural factor productivity as well as estimate the technical efficiency of crop yields in Sub-Saharan African countries. The study aims to address the following specific objectives: investigating the impacts of climate change on agricultural factor productivity, examining the impact of factor productivity on cereal crop yields, and estimating the level of technical efficiency of cereal yields in the region.
3. The literature was edited and expanded to include recent related literature. We included literature for each variable and identified gaps. The methodology section was unclear, particularly the panel data model. We are now revising the model from specification to model formulation by providing a trustworthy reasoning for its application. The study's discussion, conclusion, and policy implications have all been updated. The updated section is indicated in red colour.
4. We are free to receive comments and revised again

**Response to Reviewer #2:**

Thank you for your time and important thoughts.

1. The abstract should present quantifiable results. Here, the abstract is rewritten in quantitative terms “*The world aims to ensure environmental sustainability and increase agricultural factor productivity, yet the harsh impact of climate change coexists and remains a persistent threat.  Therefore, study aims to estimate the technical efficiency of cereal crop yields and investigate the impacts of climate change on agricultural factor productivity. For this purpose, panel data from 35 sub-Saharan African countries between 2005 and 2020 was employed. For analysis, the pooled OLS and stochastic frontier models were employed. The results revealed that in the region, the average efficiency score for producing cereal crops between 2005 and 2020 was 83%. The stochastic frontier model results showed that labour contributed 51.5% and fertilizer contributed 5.7% to raising the technical efficiency of cereal crop yields, whereas arable land per hectare reduced the technical efficiency of cereal yields by 44.7%. The pooled OLS regression result showed that climate change proxies (CO2 and methane emissions) diminish land, labour, and fertilizers productivity at a 1% significance level, whereas GDP per capita boosts significantly the total factor productivity in agriculture*. *This confirmed that agricultural methane emissions (CH4), carbon dioxide emissions (CO2) have reduced land, labour, and fertilizer input productivity. The results concluded that the region had a high level of technical efficiency; of which labour and fertilizer inputs contributed the largest share; however, their productivity has dwindled due to climate change. To increase cereal crop yield efficiency and limit the negative effects of climate change on agricultural input productivity, the region should combine skilled labour and fertilizer with sophisticated agriculture-based technologies, as well as adopt climate resistance technologies (genetically improved seed and planting revolution mechanisms).”*
2. It is preferable to present the agricultural condition in Sub-Saharan Africa as part of the introduction. We believe that your comments are valuable, and we have added literature about the significance of agriculture and how climate change affects agriculture. For example, In Sub-Saharan African (SSA) countries, agriculture contributes 40% of GDP and employs more than half of the workforce [1]. By 2050, improvements in agricultural productivity in the region will significantly offset the GDP losses due to climate change. However, in regions where irrigation will be extended, climate change is insufficient to offset GDP losses [2] . You can see the revised, and the revised parts are highlighted in red color.
3. It will be helpful to draw a research gap. The research gap also rewrite and enriched like(Studies like [5], [24], [27]–[29] focused on the impact of climate change on crop yields whereas [23], [36], [39], [40] investigated on climate change impact on technical efficiency. However, they failed to investigate the impact of climate change on agricultural factor productivity as well as estimate the technical efficiency of crop yields in Sub-Saharan African countries. The study aims to address the following specific objectives: investigating the impacts of climate change on agricultural factor productivity, examining the impact of factor productivity on cereal crop yields, and estimating the level of technical efficiency of cereal yields in the region).
5. Update the literature review to reflect each variable in the study model. The literature was generated by incorporating relevant material for each variable. The literature part of the study contains references to CO4, CO2, labour, land, and fertilizer production.
6. Please specify the models used in the results. Why did they utilize separate models? The reason for using various models is that the study has two objectives. The first goal is to examine the technical efficiency of cereal crops, and the second is to investigate the influence of climate change on input productivity. As a result, the stochastic frontier modal should be used to address the first objective, while one of the panel models can address the second. Based on the specification test of panel models, the Pooled is best model in this circumstance. The approaches are revised by justifying their reasoning using literature. See the revised, which are indicated in red color.
7. We have ensured consistency in table and figure formats, corrected issues, and reworked the entire presentation.
8. Policy recommendations and research limitations are offered as distinct subtitles following the study's conclusion.

We notify you that the authors are eager to take any comments that the reviewer believes as they should be corrected further.
