# Supplementary material for: Factor productivity impacts of climate change and estimating the technical efficiency of cereal crop yields: Evidence from sub-Saharan African countries
Source: PLoS One. 2024 Nov 21;19(11):e0310989. doi: 10.1371/journal.pone.0310989 (PMC11581406; doi:10.1371/journal.pone.0310989)
Supplement: Attachment — Submitted filename: comments.docx [file pone.0310989.s004.docx]

This manuscript titled “Factor productivity impacts of climate change and estimating the technical efficiency of cereal crop yields: Evidence from sub-Saharan African countries” estimates the technical efficiency of cereal crop yields and examines the effects of climate change on agricultural productivity in 35 sub-Saharan African countries from 2005 to 2020. Using pooled OLS and stochastic frontier models, the average efficiency score for cereal production was found to be 83%. Labour and fertilizer significantly enhanced efficiency, contributing 51.5% and 5.7%, respectively, while arable land per hectare negatively impacted efficiency by 44.7%. Climate change indicators such as CO2 and methane emissions significantly reduced the productivity of land, labour, and fertilizers, whereas GDP per capita positively influenced overall agricultural productivity. The findings highlight high technical efficiency in the region, primarily driven by labour and fertilizer inputs, but these have been adversely affected by climate change. This work is an interesting contribution, and the authors are commended for their efforts. However, in order for the manuscript to meet the standards for publication, this submission requires a major revision. Hence, the following specific revisions are necessary:

1. The introduction discusses climate change impacts and the need for climate-smart agriculture in Sub-Saharan Africa (SSA) but fails to address the diverse local contexts that influence the adoption and effectiveness of these practices. Different countries and regions face unique climatic challenges, agricultural methods, and socio-economic conditions. Consequently, more nuanced research findings and examples from specific countries or communities would enhance the understanding of how climate-smart agriculture can be tailored to diverse environments and cultural practices, the authors should please revise.
2. Still in the introduction section, which describes the challenges faced by smallholder farmers and the potential benefits of climate-smart agriculture, this section lacks a discussion of the role that governance and policy frameworks play in facilitating or hindering the adoption of such technologies, please the authors must pay attention to this gap.
3. The literature review in section two of this manuscript should be merged with introduction section and critically summarise this section.
4. All the Tables presented in this manuscript must be revised based on the standard journal requirements.
5. The authors did not add discussion on 4.1, 4.2, 4.3, 4.4 and 4.5
6. No seen interpretation on 4.6 and bad notations.
7. The grammatical structure and tenses of the manuscript should be improved.
8. Please, ensure that the line spacing in the text is consistent and in line with journal requirement. Check grammatical errors and ensure proper formatting for all text.
