# Supplementary material for: Factor productivity impacts of climate change and estimating the technical efficiency of cereal crop yields: Evidence from sub-Saharan African countries
Source: PLoS One. 2024 Nov 21;19(11):e0310989. doi: 10.1371/journal.pone.0310989 (PMC11581406; doi:10.1371/journal.pone.0310989)
Supplement: Attachment — Submitted filename: 2nd Author response to Reviewer.docx [file pone.0310989.s005.docx]

Author Response to Reviewer

*Dear reviewer, we are delighted to express appreciation for your valuable comments and the time you dedicate to provide such a very good feedbacks. We sincerely believe that the comments are constructive, scientific, and more valuable for this work. As a result, we have attempted to address the comments, and the revised sections are highlighted in red. First and foremost, we apologise for any responses that did not satisfy or address your comments.*

**Comment≠1**: The introduction discusses climate change impacts and the need for climate-smart agriculture in Sub-Saharan Africa (SSA) but fails to address the diverse local contexts that influence the adoption and effectiveness of these practices. Different countries and regions face unique climatic challenges, agricultural methods, and socio-economic conditions. Consequently, more nuanced research findings and examples from specific countries or communities would enhance the understanding of how climate-smart agriculture can be tailored to diverse environments and cultural practices, the authors should please revise.

***Our reply****:* *You have excellent insight; it is true that the introduction part only discussed the consequences of climate change and the necessity for climate-smart agriculture adoption. We endeavour to respond to your comment. As a result, we have considered the effectiveness of CSA, as well as how different countries' socioeconomic and distinctive tactics influenced adoption outcomes. As a result, we have collected contemporary literatures that support the effectiveness, and different elements influence CSA like* “In Africa, CSA was embraced as a strategy to address concerns about agricultural productivity, improve adaptation measures, and increase climate resilience. However, in most countries, it has been challenged by a lack of national Climate-Smart Agriculture Investment Plans (CSAIPs) [42], a lack of clear conceptual understanding, inadequate policies, and insufficient capital [43], [44]. It has also been affected by high initial investment costs, labour requirements, and management intensity associated with conservation agriculture and rainwater harvesting [45], gender gaps, ecological and environmental factors [46], [47], shortage of cropland, land tenure issues, lack of adequate knowledge about CSA, slow return on investment ,and insufficient policy and implementation schemes [48], [49]. Climate-smart agriculture has been mainstreamed into agricultural development plans through the construction of regional, sub-regional, and national climate change policies and strategies aimed at mitigating climate change and strengthening African people's adaptive capacity [50]. Smallholders face challenges in implementing climate-smart agriculture (CSA) due to unclear duties, insufficient links between administration levels, limited resources, and political intervention [51]”.

**Comment≠2**: Still in the introduction section, which describes the challenges faced by smallholder farmers and the potential benefits of climate-smart agriculture, this section lacks a discussion of the role that governance and policy frameworks play in facilitating or hindering the adoption of such technologies, please the authors must pay attention to this gap.

***Our reply:*** *Of course, the introduction section does not go into detail regarding the challenges and benefits of CSA, as well as how governance and policy frameworks respond to the adoption of climate smart agriculture. By accepting the comments and providing literature that can answer your queries* “African governments face political, economic, and governance problems while implementing climate-smart agriculture (CSA) to achieve the United Nations Sustainable Development Goals [52]. In most African countries, there is a lack of consideration for the replacement, complementing, or conditional effects of policy initiatives on the adoption of smart climate practices [53].The government and policy alternatives for climate smart adoption have inadequate and ineffective interventions in decision making, accessing inputs, and formulating and implementing policies in Africa [54].”

**Comment≠3**: The literature review in section two of this manuscript should be merged with introduction section and critically summarise this section.

**Our reply:** *Dear Reviewer, We investigated the published articles and found that they had a literature review parts. We have accepted your comment. Because we recognise that your idea of constructing an introduction by incorporating literature into the introductory part will undoubtedly boost the manuscript's quality. Of course, creating a sensible opening that clearly demonstrates the study's limitations would boost its quality. As a result, we rewrote and thoroughly summarised the introduction and literature parts together. Track modification highlights the introduction's summarising and combined sections.*

**Comment≠4**: All the Tables presented in this manuscript must be revised based on the standard journal requirements.

**Our reply:** *We attempted to rewrite the tables, figures, and equations based on the manuscript standard or guideline. We have also referred the published articles in the PLOS one.*

**Comment≠5**: The authors did not add discussion on 4.1, 4.2, 4.3, 4.4 and 4.5

**Our reply: We have added the discussion part for 4.1 like** “Results show that, with homogeneous technology, the estimated mean technical efficiency of 38.2% implies that, in Africa, almost 62% of the potential agricultural output is untapped [78]. Cereal production in Sub-Saharan Africa has experienced low and unpredictable growth rates due to its rain- fed nature and subsistence orientation over the last four decades [96]. In recent years, crops were planted on 98.6 million hectares, yielding 162 million tons [97].”

**For 4.2 “** In LDCs, the impacts of agricultural inputs are stumpy in influencing the growth of agriculture, but producers use cropping intensity to compensate for the dropping average yield per harvest and to assure sustainable land productivity development[59]. Organic fertilizer treatments resulted in an 11% -13% yield increase and a 4% -5% higher net economic gain [67], [68]. After the 2000s, the average annual growth rate of agricultural productivity is expected to be 3.13% for the sample counties [4].”

**For 4.3** “With the expansion of technology, CO2 increases world yields by roughly 1.8% every decade while decreasing them by 1.5% every decade [11]. Carbon dioxide (CO2), nitrogen oxide (N2O), and methane (CH4) are the most prevalent greenhouse gases (GHGS), and they are creating a variety of serious consequences in the agriculture sector [12], [13].In this study, climate change and cereal yields show a consistent and similar pattern in the majority of the region from 2005 to 2020. However, numerous Sub-Saharan African countries, including South Africa and Angola, have high carbon emissions and low cereal crop yields; in particular, South Africa's carbon emissions are disproportionately high in relation to its cereal crop yields.”

**For 4.4 and 4.5** With a technological efficiency of 38.2%, Africa is predicted to miss out on around 62% of its agricultural potential [78], particularly in Ethiopia, where the estimated TE of 66% implies possibilities for greater crop production efficiency [79]. A firm is considered technically efficient if it is able to manufacture more goods without expanding the number of production inputs. We have investigated and estimated the technical efficiency score of cereal crop production in the region. The results in Fig. 4 demonstrate that the region has an average minimum technical efficiency score of 0.683 and a maximum technical efficiency score of 0.994. This signifies that the region is highly technologically efficient.” “From 1960 to 1991, crop output in SSA increased by 2.7% each year, while food production increased by 2.4% per year. But, worker productivity declined by an average of one percent per year in SSA agriculture [98]. Crop productivity dropped between 2008 and 2019, with no evidence of improvement [99]. “Table 2” shows the average cereal crop yield per unit of input. The results confirmed that the average cereal crop yield per hectare from 2005 to 2020 was 1497.332kg. During these years, 99% of the countries in the region have produced a maximum of 7541kg per hectare. However, the productivity of inputs such as labour, land, and fertilizer use has had different effects on cereal crop yields across countries in the region.”

**Comment≠6**: No seen interpretation on 4.6 and bad notations.

**Our reply:** This section is only required for to identify does the variable is stationary or non-stationary. The results are confirmed the stationary of the data. To solve its bad notation we have stated this table in the appendix section.

**Comment≠7**: The grammatical structure and tenses of the manuscript should be improved.

Our reply: *Of course, the manuscript includes grammatical structure issues, and we attempted to resolve these issues by reviewing and rewriting the entire section. Furthermore, our senior staffs, who have experience in publishing manuscripts, have attempted to revise and fix the grammatical errors*.

**Comment≠7**: Please, ensure that the line spacing in the text is consistent and in line with journal requirement. Check grammatical errors and ensure proper formatting for all text.

***Our reply:*** *We rereading and revised very well to ensure the line spacing and text consistency problem. As far as possible, we try to prove the formatting, spacing and other required requirements****.***

***If there is any more please do note hesitate to give***
