# Supplementary material for: Factor productivity impacts of climate change and estimating the technical efficiency of cereal crop yields: Evidence from sub-Saharan African countries
Source: PLoS One. 2024 Nov 21;19(11):e0310989. doi: 10.1371/journal.pone.0310989 (PMC11581406; doi:10.1371/journal.pone.0310989)
Supplement: Attachment — Submitted filename: 2comment.docx [file pone.0310989.s006.docx]

This manuscript titled “Factor productivity impacts of climate change and estimating the technical efficiency of cereal crop yields: Evidence from sub-Saharan African countries” estimates the technical efficiency of cereal crop yields and examines the effects of climate change on agricultural productivity in 35 sub-Saharan African countries from 2005 to 2020. Using pooled OLS and stochastic frontier models, the average efficiency score for cereal production was found to be 83%. Labour and fertilizer significantly enhanced efficiency, contributing 51.5% and 5.7%, respectively, while arable land per hectare negatively impacted efficiency by 44.7%. Climate change indicators such as CO_2_ and methane emissions significantly reduced the productivity of land, labour, and fertilizers, whereas GDP per capita positively influenced overall agricultural productivity. The findings highlight high technical efficiency in the region, primarily driven by labour and fertilizer inputs, but these have been adversely affected by climate change. This work is an interesting contribution, and the authors are commended for their efforts. I wholeheartedly recommend this paper for publication with no revisions.
