# Supplementary material for: Factor productivity impacts of climate change and estimating the technical efficiency of cereal crop yields: Evidence from sub-Saharan African countries
Source: PLoS One. 2024 Nov 21;19(11):e0310989. doi: 10.1371/journal.pone.0310989 (PMC11581406; doi:10.1371/journal.pone.0310989)
Supplement: Attachment — Submitted filename: Author Response letter 3.docx [file pone.0310989.s007.docx]

**Author Response letter to Editor**

Dear editor of the PLOS ONE, we would like to thank you for your unreserved contribution. Your comments were constructive this work from the first round to until it reaches at this stages. We understand your aid is how much valuable to improve the scientific value of this manuscript. The referencing is cited using mendeley software, but it is not free from some referencing problem. As you request us to edit and make a correction on references, we try to reread, edited and replaced some old references by recent references. While we are editing the reference lists, there is problem of incomplete referencing that raised by software problem as well as use of old references. But there is no referenced source that is not listed in bibliography because all cited references are listed in the reference list section. Some retracted references replaced by recent references like “ 1) F. Muhirwa *et al.*, “Ecological balance emerges in implementing the water-energy-food security nexus in well-developed countries in Africa,” *Sci. Total Environ.*, vol. 833, 2022, doi: 10.1016/j.scitotenv.2022.154999.2) Y. Karavias and E. Tzavalis, “Generalized fixed-T panel unit root tests,” *Scand. J. Stat.*, vol. 46, no. 4, pp. 1227–1251, 2019, doi: 10.1111/sjos.12392.
